# Polyamine depletion induces G_1 _and S phase arrest in human retinoblastoma Y79 cells

**DOI:** 10.1186/1475-2867-8-2

**Published:** 2008-01-21

**Authors:** Akiko Ueda, Makoto Araie, Shunichiro Kubota

**Affiliations:** 1 Department of Ophthalmology, Graduate School of Medicine, The University of Tokyo, 7-3-1 Hongo, Bunkyo-ku, Tokyo 113-0033, Japan; 2 Department of Life Sciences, Graduate School of Arts and Sciences, The University of Tokyo, 3-8-1 Komaba, Meguro-ku, Tokyo 153-8902, Japan

## Abstract

**Background:**

Polyamines and ornithine decarboxylase (ODC) are essential for cell proliferation. DL-α-difluoromethylornithine (DFMO), a synthetic inhibitor of ODC, induces G_1 _arrest through dephosphorylation of retinoblastoma protein (pRb). The effect of DFMO on cell growth of pRb deficient cells is not known. We examined the effects of DFMO on pRb deficient human retinoblastoma Y79 cell proliferation and its molecular mechanism.

**Methods:**

Using cultured Y79 cells, the effects of DFMO were studied by using polyamine analysis, western blot, gel shift, FACS and promoter analysis.

**Results:**

DFMO suppressed the proliferation of Y79 cells, which accumulated in the G1 and S phase. DFMO induced p27/Kip1 protein expression, p107 dephosphorylation and accumulation of p107/E2F-4 complex in Y79 cells.

**Conclusion:**

These results indicate that p107 dephosphorylation and accumulation of p107/E2F-4 complex is involved in G_1 _and S phase arrest of DFMO treated Y79 cells.

## Background

The polyamines, spermidine, spermine, and their precursor putrescine are essential for cell growth and the regulation of the cell cycle [1,2]. Many reports describe increased polyamine and ODC levels in various cancers [3-7]. Intracellular polyamine levels are regulated and primarily depend on the activity of ornithine decarboxylase (ODC), which catalyzes the first rate-limiting step in polyamine biosynthesis [8]. Depletion of polyamines by DL-α-difluoromethylornithine (DFMO), a specific inhibitor of ODC, has been reported to inhibit growth of various kinds of cells [3,4,9-11]. DFMO induces expression of CDK inhibitors such as p21 and p27 [12-14] and G_1 _arrest associated with hypophosphorylation of pRb [13].

P16, one of the major p16 CDK inhibitor family, competes with cyclin D to bind with CDK4 and CDK6, and the both p21 and p27 which are p21 CDK inhibitor family associate with cyclin/CDK complexes including CDK2, 4 and 6. It is established that increased expression of p16, p21 or p27 suppreses CDK activities, which leads to cell cycle arrest [15,16].

The pRb protein and E2F are thought to be a critical component in the control of the restriction point of the G_1_/S transition of the cell cycle [17]. Free E2F activates E2F-dependent transcription of genes required for S-phase entry. E2F/pRb complex represses transcriptional activity of E2F [18]. When pRb protein is phosphorylated by cyclin CDK complexes, the E2F/pRb complex is disrupted, and the released E2Fs from pRb lead to activation of E2F target genes [17,18]. p107 and p130 are similar to pRb in their structures and functions [18,19]. E2F-1, E2F-2, and E2F-3 bind almost exclusively to pRb; E2F-4 binds to p107 and p130 with high affinity, and also associates with pRb in some cell types; E2F-5 associates with p130 [18].

Overexpression experiments have revealed that each of pRb family proteins can induce G_1 _cell cycle arrest [20-23]. Recently, it has been reported that p107 blocks cell cycle inside S phase in addition to G_1 _arrest [24].

Since little is known about effects of DFMO on cell growth of pRb deficient cells, we examined whether DFMO has an anti-proliferative effect and how DFMO affects cell cycle in human retinoblastoma Y79 cells that lack functional pRb protein.

## Results

### Inhibition of Y79 cell growth and induction of G_1 _and S phase arrest by DFMO

**Figure 1:**
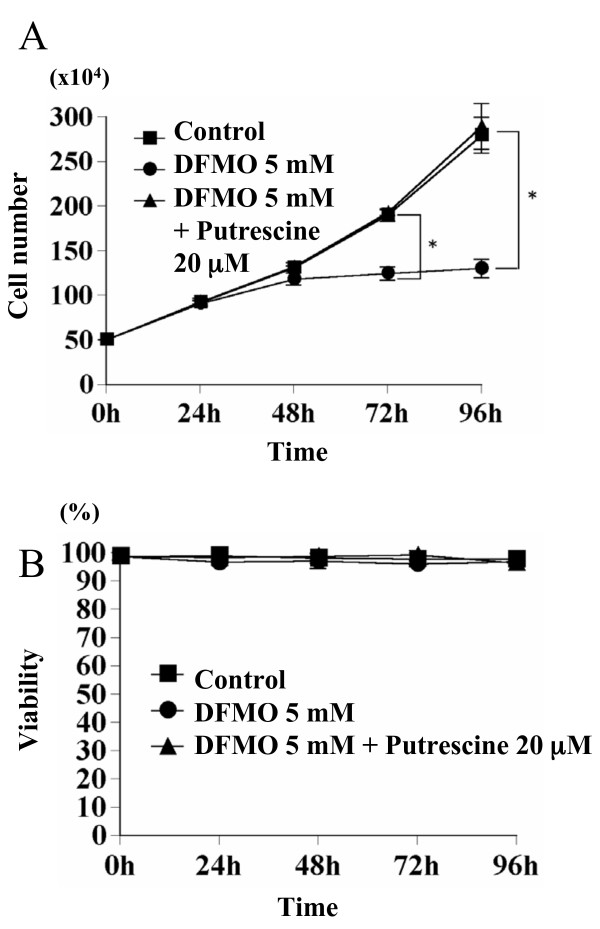
**Effects of DFMO on Y79 cell growth**. A: Y79 cells were treated with 5 mM DFMO over a 96 h period, and cell number was counted. B: Cell viability was analyzed with trypan blue dye exclusion test. Significantly different compared to 0 h: *p < 0.01

Preliminary experiment showed that DFMO (1–5 mM) inhibited Y79 cell growth in a dose-dependent manner (not shown). Therefore, we used 5 mM DFMO in the present study. The effects of 5 mM DFMO on Y79 cell growth were studied. After 72 h and 96 h, 5 mM DFMO inhibited Y79 cell growth 34.5 ± 3.97 and 53.5 ± 3.7%, respectively, compared to control without DFMO treatment (p < 0.001) (Fig. 1A). The growth suppression by 5 mM DFMO was completely restored by addition of 20 mM putrescine, indicating the specificity of the effect of DFMO on cell growth and the involvement of polyamines in Y79 cell growth. Viabilities of the cells treated with 5 mM DFMO were 96.6 ± 1.08 %, 96.67 ± 2.65 %, 95.88 ± 1.32 %, and 96.83 ± 1.58 % after 24 h, 48 h, 72 h, and 96 h respectively (not significant, compared to that observed at 0 h) (Fig. 1B). The results suggest that DFMO did not induce Y79 cell death. We next analyzed the effect of DFMO on ODC activities and polyamine levels in cells. DFMO (5 mM) treatment decreased ODC activities 96.7 ± 0.81 % and 99.6 ± 0.01 at 24 h and 48 h, respectively, compared to that observed at 0 h (p < 0.001) (Table 1). The levels of putrescine, spermidine, and spermine were significantly reduced at 24 h and 48 h (Table 2).

**Table 1:** ODC activities of Y79 cells treated with DFMO

0 h	24 h	48 h	
13.33 ± 1.17	0.66 ± 0.01*	0.05 ± 0.001*	(nmolCO_2_/h/mg protein)

Significantly different compared to 0 h: *p < 0.001

**Table 2:** Polyamine levels of Y79 cells treated with DFMO

	Putrescine	Spermidine	Spermine	
0 h	1643.7 ± 143.2	1014.1 ± 235.1	659.3 ± 96.2	
24 h	90.5 ± 22.7*	597.7 ± 118.2**	405.7 ± 102.9***	
48 h	45.8 ± 9.5*	221.7 ± 87.6**	422.8 ± 87.5***	(nmol/10^5 ^cells)

Significantly different compared to 0 h

*p < 0.0001, **p < 0.002, ***p < 0.03

**Figure 2:**
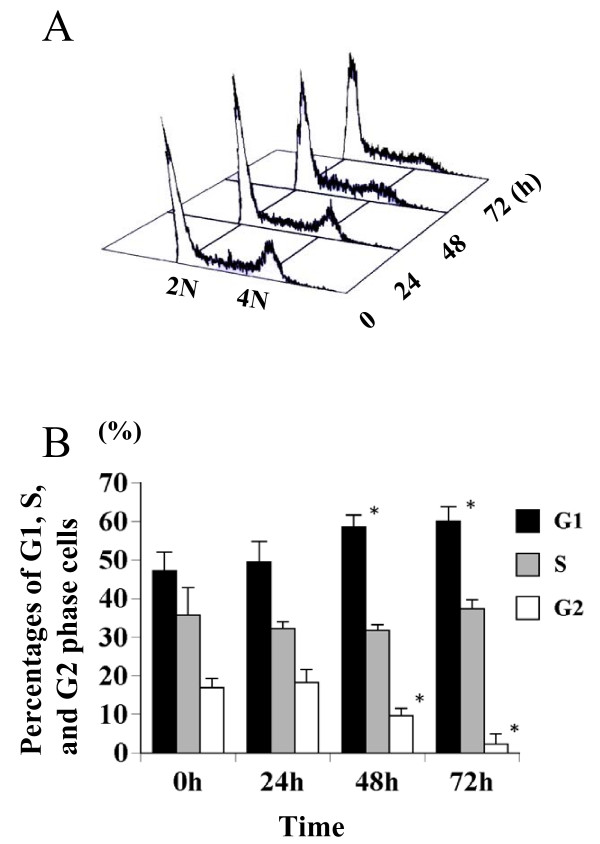
**Effects of DFMO on Y79 cell cycle**. Y79 cells were treated with 5 mM DFMO for 24, 48, and 72 h. Cell cycle was analyzed using a flow cytometer (A), as described under Experimental Procedures, and percentages of G_1_, S, and G_2 _phase cells are shown (B). Significantly different compared to 0 h: *p < 0.01

Cell cycle analysis was done using a flow cytometer. As shown in Fig. 2A and 2B, the percentages of G_1 _phase cells were significantly increased at 48 h and 72 h. The percentages of G_2 _phase cells were significantly reduced at 48 h and 72 h. These data suggest that DFMO treatment blocked cell cycle inside S phase in addition to G_1 _arrest.

### Effects of polyamine depletion on expression of CDK inhibitors and pRb family proteins

**Figure 3:**
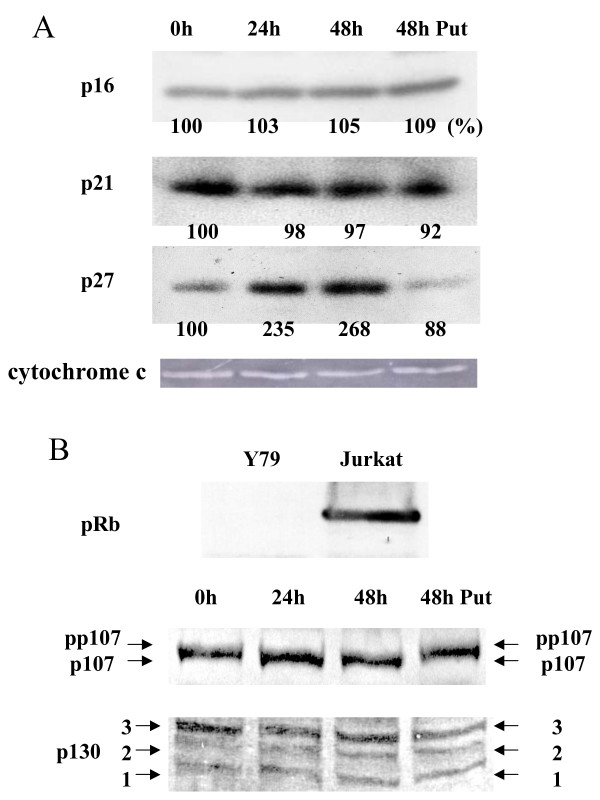
**Expression of CDK inhibitors and pRb family proteins**. A: Y79 cells were treated with 5 mM DFMO for 24 and 48 h. Cell lysates (100 μg) were subjected to western blot analysis using the antibodies against p16, p21, p27, and cytochrome c (as a control). Quantification of the bands was performed using the NIH image software, and corrected using the levels of cytochrome c. The data were expressed as %, compared to that observed at 0 h (100%). The experiments were repeated twice and similar results were obtained. A typical result is presented. B: Cell lysates (100 μg) of Y79 cells and Jurkat cells (positive control) were subjected to western blot analysis using the antibody against pRb (upper panel). Y79 cells were treated with 5 mM DFMO for 24 and 48 h. Cell lysates (50 μg) were subjected to western blot analysis using the antibodies against p107 and p130 (middle and lower panels). Signals were visualized using ECL. pp107 and p107 indicate hyperphosphorylated p107 and hypophosphorylated p107, respectively. 1, 2, and 3 indicate phosphorylated forms of p130.

To study the mechanism of S and G_1 _phase arrest, we studied the effect of DFMO on the expression of CDK inhibitors and pRb family proteins. The expression of p27 was markedly induced by DFMO treatment. The magnitude of induction at 24 h and 48 h was 235% and 268%, respectively, compared to that at 0 h (100%). Addition of putrescine rescued the effect of DFMO. In contrast, p16 and p21 were not significantly changed (Fig. 3A). We confirmed that Y79 cells did not express pRb using western blot analysis (Fig. 3B), and examined the expression of p107 and p130 proteins. Hypophosphorylated forms of p107 accumulated at 24 h and 48 h after DFMO treatment (Fig. 3B). On the contrary, the expression levels of each form of p130 protein did not change until 48 h after DFMO treatment (Fig. 3B).

### Polyamine depletion increased p107/E2F-4 complex

**Figure 4:**
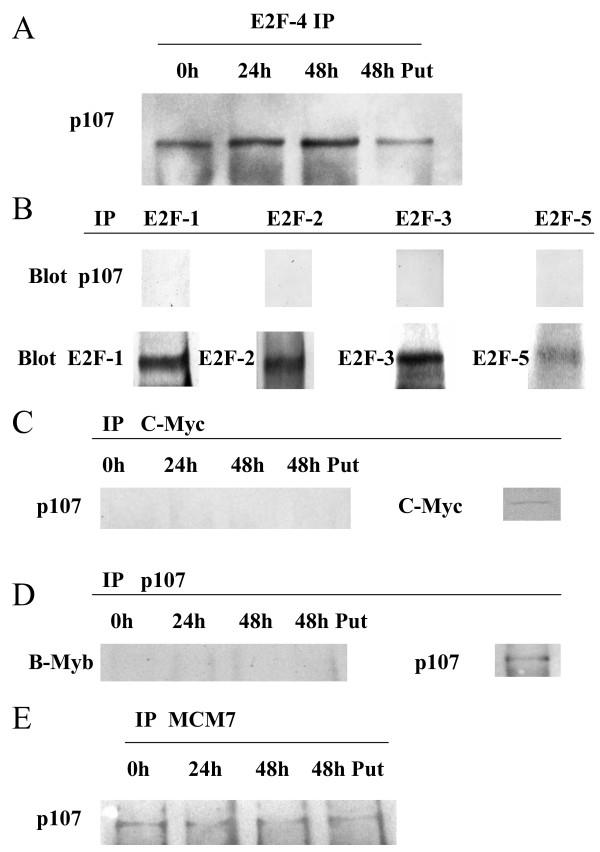
**Effects of DFMO on association of p107 and cellular proteins**. A: Effects of DFMO on p107/E2F complexes in Y79 cells. Whole cell lysates (100 μg) treated with 5 mM DFMO for 0, 24, 48 h or 5 mM DFMO and 20 μM putrescine for 48 h were immunoprecipitated with anti E2F-4 antibody and immunoblotted with anti p107 anitibody. B: Cell lysates (100 μg) were immunoprecipitated with anti E2F-1, 2, 3, and 5 antibodies and immunoblotted with anti p107 anitibody (upper panels) or with anti E2F-1, 2, 3, and 5 antibodies (lower panels). C: Cell lysates (100 μg) were immunoprecipitated with anti C-Myc antibody and immunoblotted with anti p107 anitibody (left panel). The lysate of Y79 cells treated with DFMO for 24 h were immunoprecipitated with anti C-Myc antibody and immunoblotted with anti C-Myc antibody (right panel). D: Cell lysates (100 μg) were immunoprecipitated with anti p107 antibody and immunoblotted with anti B-Myb anitibody (left panel). The lysate of Y79 cells treated with DFMO for 24 h were immunoprecipitated with anti p107 antibody and immunoblotted with anti p107 antibody (right panel). E: Cell lysates (100 μg) were immunoprecipitated with anti MCM7 antibody and immunoblotted with anti p107 anitibody.

Since p107 is known to be associated with E2F-4 [24,25], C-Myc [26,27], and B-Myb [28], and suppress their transcriptional activities, we investigated whether DFMO induced binding of these proteins with p107. Whole cell extracts (100 μg protein) derived from cells treated with DFMO for 0 h, 24 h, and 48 h were immunoprecipitated with antibody against E2F-4 or C-Myc. Immunoprecipitated proteins were analyzed by western blotting using anti p107 antibody. The p107/E2F-4 complex was detected at 0 h, and DFMO treatment induced 1.6 ± 0.2- and 1.8 ± 0.2-fold increase in amount of the complex at 24 h and 48 h, respectively, compared to 0 h (p < 0.01) (Fig. 4A). Putrescine (20 μM) blocked the increase of the amount of p107/E2F-4 complex, indicating the specificity of the effect of DFMO on binding of E2F-4 with p107. Other E2F family proteins (E2F1, 2, 3, and 5) were detected in DFMO treated Y79 cells (Fig. 4B, lower panel), but the complex between p107 and these proteins were not detected (Fig. 4B, upper panel). Although C-Myc was detected in DFMO treated Y79 cells (Fig. 4C, right panel), the complex of p107 and c-Myc was not detected (Fig. 4C, left panel). Whole cell extracts (100 μg protein) derived from cells treated with DFMO for 0 h, 24 h, and 48 h were immunoprecipitated with antibody against p107. Immunoprecipitated proteins were analyzed by western blotting using anti B-Myb antibody and the complex of p107 and B-Myb was not detected (Fig. 4D, left panel). It has been reported that pRb and pRb family protein can directly regulate DNA replication by their association with MCM7/CDC47 [29]. Therefore, we analyzed the MCM7-immunoprecipitated whole cell extracts by western blotting using anti p107 antibody. The complex of p107 and MCM7 was detected, but it was not increased by DFMO treatment (Fig. 4E).

### p107/E2F-4 complex binds to E2F binding site

**Figure 5:**
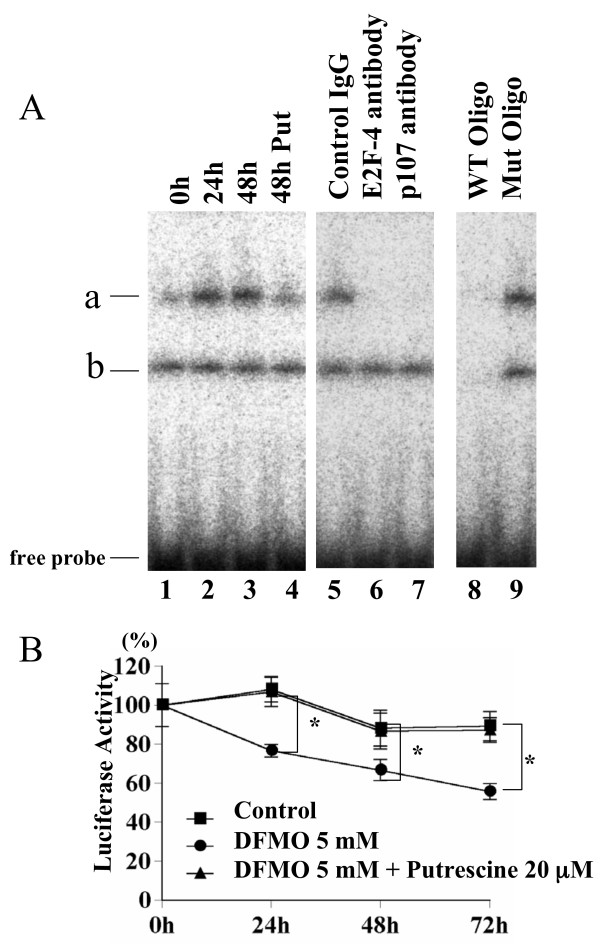
**Effect of DFMO on E2F transcriptional activity**. A: E2F electoromobility shift assays using 10 μg of nuclear extracts from DFMO treated Y79 cells and E2F binding oligonucleotides. Lanes 1–4 : Nuclear extracts from Y79 cells treated with 5 mM DFMO for 0, 24, 48 h or 5 mM DFMO and 20 μM putrescine for 48 h were incubated as described under Experimental. Lanes 5–7 : Nuclear extracts from Y79 cells treated with 5 mM DFMO for 24 h were preincubated with control IgG, E2F-4 antibody, and p107 antibody, respectively. Lanes 8, 9 : Nuclear extracts from Y79 cells treated with 5 mM DFMO for 24 h were incubated with 50 ng of unlabeled competitor (WT and MT, respectively). B: One μg of pE2WTx4-Luc and 10 ng of pRL-CMV were co-transfected into Y79 cells (5 × 10^5^) using FuGene™ Transfection Reagent. After 24 h culture media were changed to RPMI 1640 media with or without 5 mM DFMO or 5 mM DFMO and 20 μM putrescine. Cell lysates were prepared at 24 h, 48 h and 72 h after the addition of DFMO, and examined for luciferase activity to monitor the promoter activity. Significantly different compared to control cells: *p < 0.02

To clarify whether p107 is involved in repression of E2F by DFMO we performed gel shift assay using E2F consensus oligonucleotides. A fast migrating complex (Fig. 5A, b), and a complex migrating at slower rate (Fig. 5A, a) were apparent in DFMO treated Y79 cells. Complex b did not change significantly by DFMO treatment. The magnitudes of increase of complex a were 2.4 ± 0.3- and 2.4 ± 0.5-fold at 24 h and 48 h, respectively, compared to 0 h (p < 0.01) (Fig. 5A lanes 2 and 3). The binding activity in these complexes was due to E2F, as it was competed out by excess unlabeled wild type E2F consensus binding site oligonucleotides, but not by their mutants (Fig. 5A lanes 8 and 9). The addition of specific antibodies against E2F-4 and p107 to the reaction mixtures confirmed that the complex a contains E2F-4 and p107 (Fig. 5A lanes 6 and 7).

### Effect of DFMO on E2F transcriptional activity

To further clarify whether p107 is involved in transcriptional repression of E2F by DFMO we performed reporter assay using artificially synthesized four tandem repeats (E2WTx4) of the adenovirus E2 enhancer, which has two typical E2F-binding sites [30]. The plasmids, pE2WTx4 and pRL-CMV were cotransfected into Y79 cells. After 24 h culture media were changed to RPMI 1640 media with or without 5 mM DFMO or 5 mM DFMO and 20 μM putrescine. Cell lysates were prepared at indicated times after the addition of DFMO, and examined for luciferase activity to monitor the promoter activity. Luciferase activity was reduced by DFMO treatment to 76.7 ± 3.3%, 66.7 ± 5.5%, 55.7 ± 4.1% at 24 h, 48 h, and 72 h, respectively, compared to control (p < 0.02) (Fig. 5B). The addition of 20 μM putrescine completely restored the luciferase activity to the level of control, indicating the specificity of DFMO effect on E2F promoter activity.

## Discussion

Our results demonstate that DFMO has anti-proliferative effects even in pRb deficient cells, and suggest that p107 may play an important role in cell cycle control of Y79 cells.

There are several reports that DFMO induces G_1 _arrest in various kinds of cells [12-14]. It was reported that pRb is dephosphorylated by DFMO treatment [13]. Induction of CDK inhibitors that regulate pRb phosphorylation has been reported in cells treated with DFMO [12-14]. However, it is unknown about the effect of DFMO on growth and cell cycle of cells that lacks pRb expression. In this study we showed for the first time that p107 is involved in cell cycle control in response to polyamine depletion in Y79 cells.

In the present study we showed that expression of p27, but not p16 and p21, was increased after DFMO treatment. As it is established that p27 suppresses CDK activities and leads to cell cycle arrest [15,16], it is reasonably assumed that increased expression of p27 by DFMO suppressed CDK activities, which led to cell cycle arrest in the present study.

p107 plays an important role in cell cycle control by making complex with E2F-4 [24,25], c-Myc [26,27], B-Myb [28] and MCM7 [29]. When E2F/p107 complex binds to promoters, p107 recruits histone deacetylase complexes, which repress promoter activity [30-34]. Binding of E2F-4 to p107 was reported to protect E2F-4 from degradation, and this may contribute to the maintenance of active transcriptional repression in quiescent cells [35]. It was reported that p107 act as a tumor suppressor function in pRb deficient mouse [36]. Recently, the unique role of p107 inhibiting S phase progression of cells that have already passed the G_1 _restriction point receives much attention [24,37,38]. Kondo et al. reported the involvement of p107 in the inhibition of S phase progression in response to DNA-damaging agent [37]. Our data suggest that p107 is involved in not only S phase arrest but also G_1 _phase arrest in DFMO-treated Y79 cells by binding with E2F-4.

Although the mechanism of accumulation of hypophosphorylated forms of p107 remains to be elucidated, CDK inhibitors may be the most possible regulators, as p107 contains multiple CDK phosphorylation sites [18]. There may be other factors that control cell cycle. However, since targeted disruption of three Rb-related genes leads to loss of cell cycle control [25], pRb family proteins are suggested to be involved in downstream pathway of cell cycle control. Therefore, p107 and p130 may be principal factors in controlling cell cycle in pRb deficient cells.

In conclusion, we report here that DFMO induced G_1 _and S phase arrest of Y79 cells and the cell cycle arrest was mediated through dephosphorylation of p107 and accumulation of p107/E2F-4 complex which repress E2F transcriptional activity.

## Methods

### Cells and reagents

The human retinoblastoma cell line Y79 and the human T cell lymphoma cell line Jurkat were obtained from the RIKEN cell bank (Tsukuba, Japan). Cells were cultured in RPMI 1640 medium with 10% fetal bovine serum (FBS) (JRH Biosciences, Lenexa, KS) in humidified air with 5 % CO_2 _at 37°C. DFMO was kindly provided by Dr. P. McCann, Merrell Dow Research Center. The antibodies against p16, p27, p107, p130, E2F-1, E2F-2, E2F-4, E2F-5, B-Myb, cytochrome c, and c-Myc were purchased from Santa Cruz Biotechnology (Santa Cruz, CA). The antibody against p21 was obtained from BD Transduction Laboratories (Franklin Lakes, NJ). The antibodies against E2F-3 and MCM7 were obtained from Neo Markers (Fremont, CA). Propidium iodide and protein A-sepharose CL4B are obtained from Sigma. The antibody against pRb was obtained from New England BioLabs (Beverly, MA). The wild and mutant E2F oligonucleotides were obtained from Santa Cruz Biotechnology. [γ-^32^P]ATP and T4 polynucleotide kinase were purchased from Amersham Biosciences (Tokyo, Japan). The reporter plasmid pE2WTx4-Luc was kindly provided by Dr. Kiyoshi Ohtani [38]. Dual luciferase reporter assay system and pRL-CMV vector were purchased from Promega (Madison, WI). FuGene™6 Transfection Reagent was purchased from Roche.

### General experimental protocols

Cells (10^5 ^cells/ml) were treated with or without 5 mM DFMO, or 5 mM DFMO and 20 μM putrescine for the indicated times. At 0 h, 24 h, 48 h, 72 h, and 96 h, the cells in each treatment group were collected. Viable cells were counted using trypan blue exclusion method.

### ODC enzyme assay and polyamine analysis

ODC activity was determined using DL- [l-^14^C]-ornithine as a substrate, as described previously [39]. Polyamine analysis was determined using high-performance liquid chromatography (HPLC) as described [40].

### Cell cycle analysis

At 0 h, 24 h, 48 h, and 72 h, the cells were collected and washed twice with PBS and fixed with chilled 70 % ethanol on ice for 30 minutes, treated with RNase A (0.5 mg/ml) for 20 minutes at 37°C, and stained with 50 mg/ml propidium iodide for 30 minutes at 4°C. Stained cells were analyzed using EPICS XL flow cytometer (Beckman Coulter, Tokyo, Japan) and WinCycle software (Phoenix Flow Systems, Inc, San Diego, CA).

### Immunoprecipitation and western blot analysis

For immunoprecipitation, whole cell extracts (100 μg protein) were incubated at 4°C for 16 h with specific antibodies prebound to protein A-sepharose CL-4B prewashed in IP buffer (50 mM HEPES, pH 7.4, containing 150 mM NaCl, 10 mM EDTA, 100 mM NaF, and 2 mM sodium orthovanadate). For western blotting, extracted proteins (100 μg) were separated by SDS polyacrylamide gel electrophoresis, transferred to nitrocellulose membrane, probed with diluted antibody (1:1,000), and visualized by ECL (Amersham Biosciences). Quantification of the bands was performed using NIH image Version 1.62 (Wayne Rasband, National Institutes of Health, U.S.A). The protein concentration was measured using a Bradford assay kit (Bio-Rad, Hercules, CA).

### Electrophoretic mobility shift assays

At 0 h, 24 h, and 48 h, the cells were collected and washed twice with PBS. Nuclear extracts were prepared as described [41]. The sequences of the E2F oligonucleotides used were 5'-ATTTAAGTTTCGCGCCCTTTCTCAA-3' (wild) and 5'-ATTTAAGTTTCGATCCCTTTCTCAA-3' (mutant). The wild type oligonucleotides were labeled with [γ-^32^P] ATP and T4 polynucleotide kinase. Ten μg of nuclear extracts were incubated for 20 min at room temperature in 12.5 mM HEPES buffer (pH 7.9), containing 100 mM KCl, 10% glycerol, 0.1 mM EDTA, 0.75 mM DTT, 0.2 mM phenylmethylsulfonyl floride, 3 μg of poly (dI-dC), and ^32^P-labeled E2F probe (10,000 cpm). For competition, 50 ng of unlabeled competitor was added. For antibody studies, extracts were preincubated with 4 μg of antibody for 20 min at room temperature. The reaction mixtures were seperated on 6 % nondenaturing polyacrylamide (37.5:1) gels. The gels were autoradiographed using Fujix Bio Imaging Analyzer BAS2000 (Fujifilm, Tokyo, Japan).

### Transient transfection and promoter assay

One μg of pE2WTx4-Luc and 10 ng of pRL-CMV was co-transfected into Y79 cells (5 × 10^5^) using FuGene™6 Transfection Reagent according to the manufacturer's instruction (Roche). After 24 h culture media were changed to RPMI 1640 media with or without 5 mM DFMO, or 5 mM DFMO and 20 μM putrescine. Promoter activities were analyzed by dual luciferase reporter assay system according to the manufacturer's protocol (Promega).

### Statistical analysis

Data from two independent experiments performed in triplicate are presented as mean ± SD unless otherwise indicated. Statistical analysis was performed using analysis of variance, with comparison of different groups by Fisher's partial least-squares difference (PLSD) test (Statview 4; Abacus Concepts, Berkeley, CA). The P value less than 0.05 was considered to be significant.

## Authors' contributions

AY did all of the experiments. MA and SK showed and helped how to do the experiments side by side. SK organized whole the experiment. Three authors read the final version of the manuscript.
